# Supplementary material for: Insight-HXMT observations of jet-like corona in a black hole X-ray binary MAXI J1820+070
Source: Nat Commun. 2021 Feb 15;12:1025. doi: 10.1038/s41467-021-21169-5 (PMC7884741; doi:10.1038/s41467-021-21169-5)
Supplement: Supplementary file 1 — Supplementary Information [file 41467_2021_21169_MOESM1_ESM.pdf]

Supplementary Information:

Insight-HXMT observations of jet-like corona in a black hole X-ray binary MAXI J1820+070

Bei You[1,2], Yuoli Tuo[3,4], Chengzhe Li[1], Wei Wang[1,2,*], Shuang-Nan Zhang[3,4,*], Shu Zhang[3], Mingyu Ge[3], Chong Luo[1,2], Bifang Liu[5,6], Weimin Yuan[5,6], Zigao Dai[7], Jifeng Liu[8,6,9], Erlin Qiao[5,6], Chichuan Jin[5,6], Zhu Liu[5,6], Bozena Czerny[10], Qingwen Wu[11], Qingcui Bu[3,12], Ce Cai[3,4], Xuelei Cao[3], Zhi Chang[3], Gang Chen[3], Li Chen[13], Tianxiang Chen[3], Yibao Chen[14], Yong Chen[3], Yupeng Chen[3], Wei Cui[15], Weiwei Cui[3], Jingkang Deng[14], Yongwei Dong[3], Yuanyuan Du[3], Minxue Fu[14], Guanhua Gao[3,4], He Gao[3,4], Min Gao[3], Yudong Gu[3], Ju Guan[3], Chengcheng Guo[3,4], Dawei Han[3], Yue Huang[3], Jia Huo[3], Shumei Jia[3], Luhua Jiang[3], Weichun Jiang[3], Jing Jin[3], Yongjie Jin[16], Lingda Kong[3,4], Bing Li[3], Chengkui Li[3], Gang Li[3], Maoshun Li[3], Tipei Li[3,4,15], Wei Li[3], Xian Li[3], Xiaobo Li[3], Xufang Li[3], Yanguo Li[3], Zhengwei Li[3], Xiaohua Liang[3], Jinyuan Liao[3], Congzhan Liu[3], Guoqing Liu[14], Hongwei Liu[3], Xiaojing Liu[3], Yinong Liu[16], Bo Lu[3], Fangjun Lu[3], Xuefeng Lu[3], Qi Luo[3,4], Tao Luo[3], Xiang Ma[3], Bin Meng[3], Yi Nang[3,4], Jianyin Nie[3], Ge Ou[17], Jinlu Qu[3], Na Sai[3,4], Rencheng Shang[14], Liming Song[3,4], Xinying Song[3], Liang Sun[3], Ying Tan[3], Lian Tao[3], Chen Wang[5,4], Guofeng Wang[3], Juan Wang[3], Lingjun WANG[3], Wenshuai Wang[17], Yusa Wang[3], Xiangyang Wen[3], Baiyang Wu[3,4], Bobing Wu[3], Mei Wu[3], Guangcheng Xiao[3,4], Shuo Xiao[3,4], Shaolin Xiong[3], Yupeng Xu[3,4], Jiawei Yang[3], Sheng Yang[3], Yanji Yang[3], Qibin Yi[3,18], Qianqing Yin[3], Yuan You[3,4], Aimei Zhang[3], Chengmo Zhang[3], Fan Zhang[3], Hongmei Zhang[18], Juan Zhang[3], Tong Zhang[3], Wanchang Zhang[3], Wei Zhang[4], Wenzhao Zhang[13], Yi Zhang[3], Yifei Zhang[3], Yongjie Zhang[3], Yue Zhang[3,4], Zhao Zhang[14], Ziliang Zhang[3], Haisheng Zhao[3], Xiaofan Zhao[3,4], Shijie Zheng[3], Dengke Zhou[3,4], Jianfeng Zhou[16], Yuxuan Zhu[3,19], and Yue Zhu[3]

[1]School of Physics and Technology, Wuhan University, Wuhan 430072, People's Republic of China;

[2]Astronomical Center, Wuhan University, Wuhan 430072, People's Republic of China

[3]Key Laboratory of Particle Astrophysics, Institute of High Energy Physics, Chinese Academy of Sciences, Beijing 100049, People's Republic of China

[4]University of Chinese Academy of Sciences, Chinese Academy of Sciences, Beijing 100049, People's Republic of China

[5]Key Laboratory of Space Astronomy and Technology, Chinese Academy of Sciences, Beijing 100012, China
[6]School of Astronomy and Space Sciences, University of Chinese Academy of Sciences, 19A Yuquan Road, Beijing, 100049, People's Republic of China
[7]School of Astronomy and Space Science, Nanjing University, Nanjing 210023, People's Republic of China
[8]Key Laboratory of Optical Astronomy, National Astronomical Observatories, Chinese Academy of Sciences, Beijing 100101, China
[9]WHU-NAOC Joint Center for Astronomy, Wuhan University, Wuhan, Hubei 430072, China
[10]Center for Theoretical Physics, Polish Academy of Sciences, Al. Lotnikow 32/46, 02-668 Warsaw, Poland
[11]School of Physics, Huazhong University of Science and Technology, Wuhan 430074, China
[12]Tuebingen University
[13]Department of Astronomy, Beijing Normal University, Beijing 100088, People's Republic of China
[14]Department of Physics, Tsinghua University, Beijing 100084, People's Republic of China
[15]Department of Astronomy, Tsinghua University, Beijing 100084, People's Republic of China
[16]Department of Engineering Physics, Tsinghua University, Beijing 100084, People's Republic of China
[17]Computing Division, Institute of High Energy Physics, Chinese Academy of Sciences, 19B Yuquan Road, Beijing 100049,People's Republic of China
[18]School of Physics and Optoelectronics, Xiangtan University, Yuhu District, Xiangtan, Hunan, 411105, China
[19]College of Physics, Jilin University, No.2699 Qianjin Street, Changchun City, 130012, China

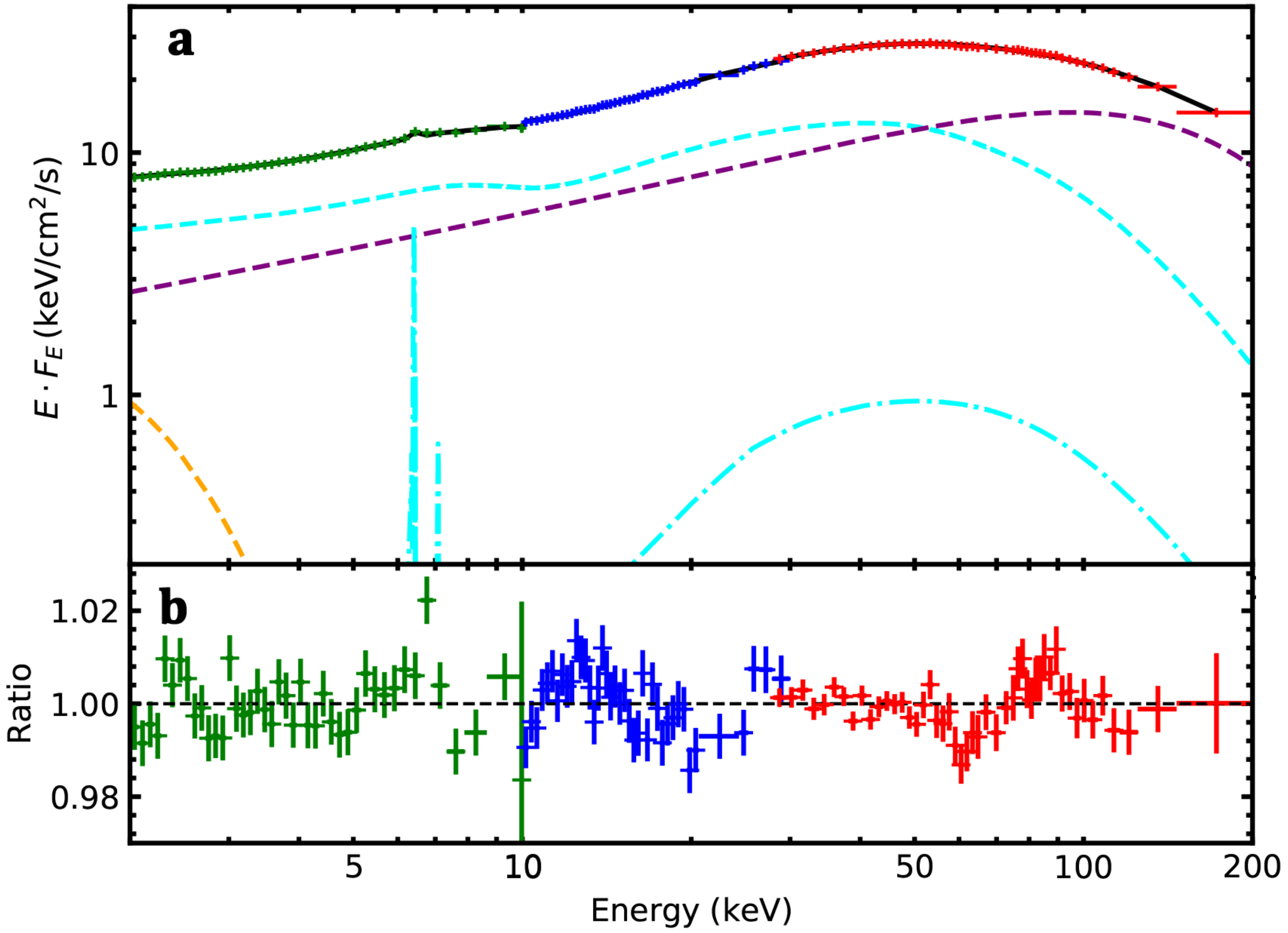

Supplementary Figure 1. Unfolded Insight-HXMT spectrum obtained on March 27, 2018 (MJD 58204, ObsID=P0114661006) with the best-fitting of TBabs∗(diskbb + relxillCp + xillverCp)∗constant model (top) and the data/model ratio (bottom). The spectrum consists of the data from LE (green), ME (blue) and HE (red) spectrum. The black dashed line is the best-fit spectrum, which is decomposed into the diskbb (orange), the Comptonization (dashed line in purple) and the reflection (dashed line in cyan) from the relxillCp component, and the reflection (dot-dashed line in cyan) from the xillverCp component. The source of uncertainties in Supplementary Figure 1b arises from the statistical uncertainties.

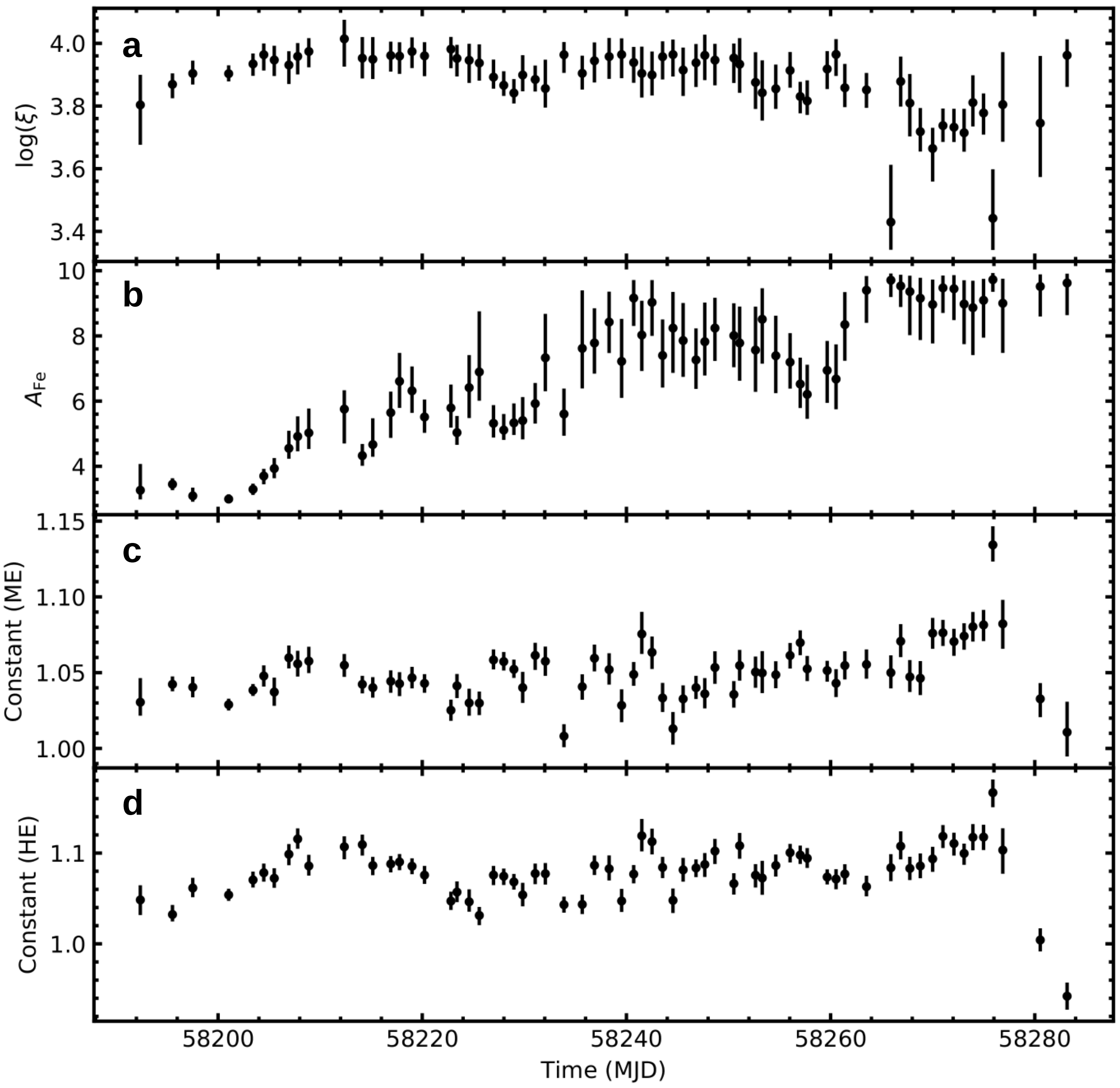

Supplementary Figure 2. Time-evolutions of the ionization parameter $\log(\xi)$ (in units of $\mathrm{erg\,cm\,s^{-1}}$), the iron abundance with respect to the solar value A_{Fe}, the constant factor of ME instrument and the constant factor of HE instrument, are plotted from top to bottom panel, respectively. The black points correspond to the median of the values and the error bars correspond to 68% confidence interval, which is calculated using the corner[1] package to analyse the probability distributions derived from the MCMC chains. The uncertainties of the fitted parameters arise from both the statistical and systematic uncertainties.

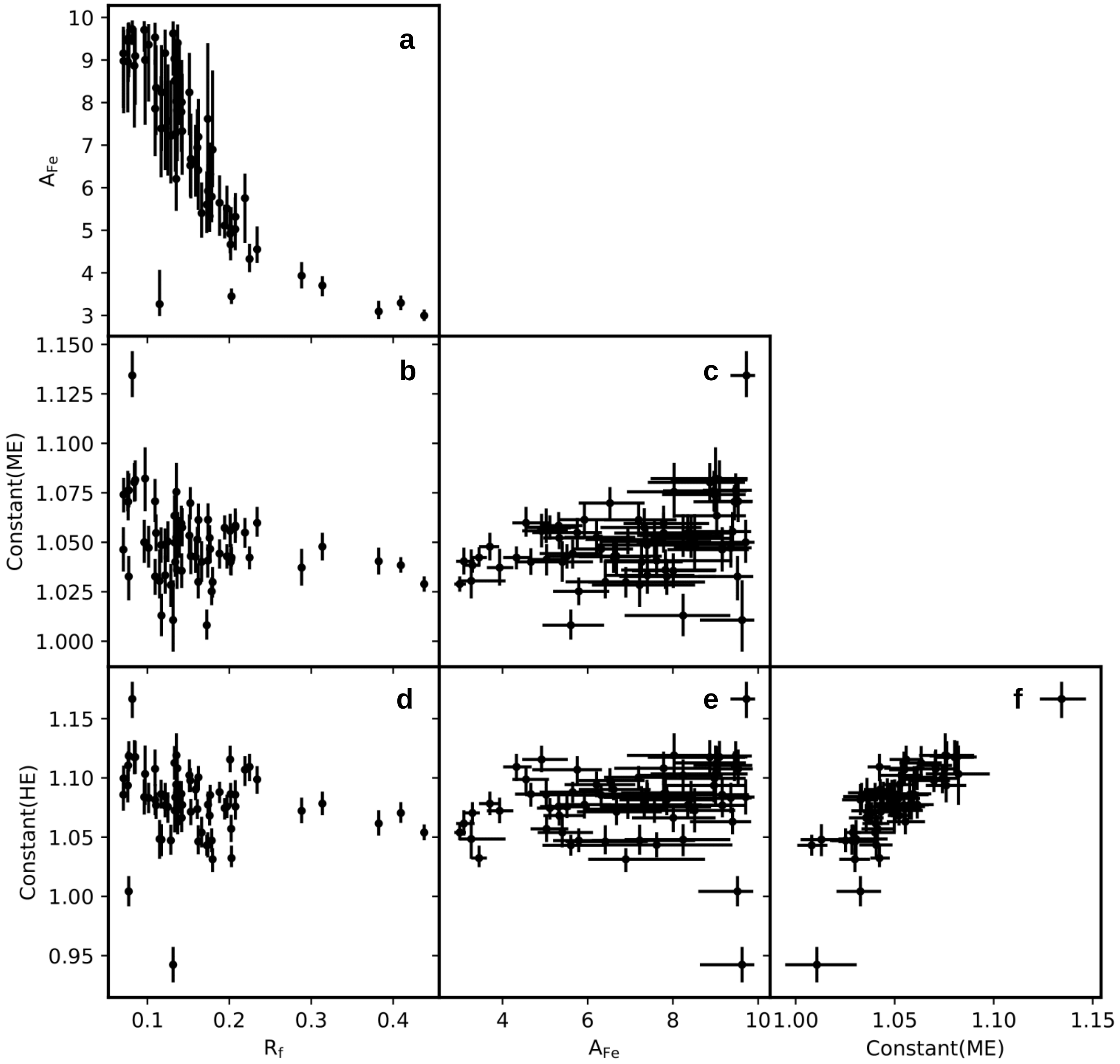

Supplementary Figure 3. The correlations of the reflection fraction of the relxillCp, the iron abundance A_{Fe} with respect to the solar abundance value, the constant factor of ME instrument and the constant factor of HE instrument, with respect to each other, which are investigated with the Spearman's rank test. The black points correspond to the median of the values and the error bars correspond to 68% confidence interval, which is calculated using the corner[1] package to analyse the probability distributions derived from the MCMC chains. The uncertainties of the fitted parameters arise from both the statistical and systematic uncertainties.

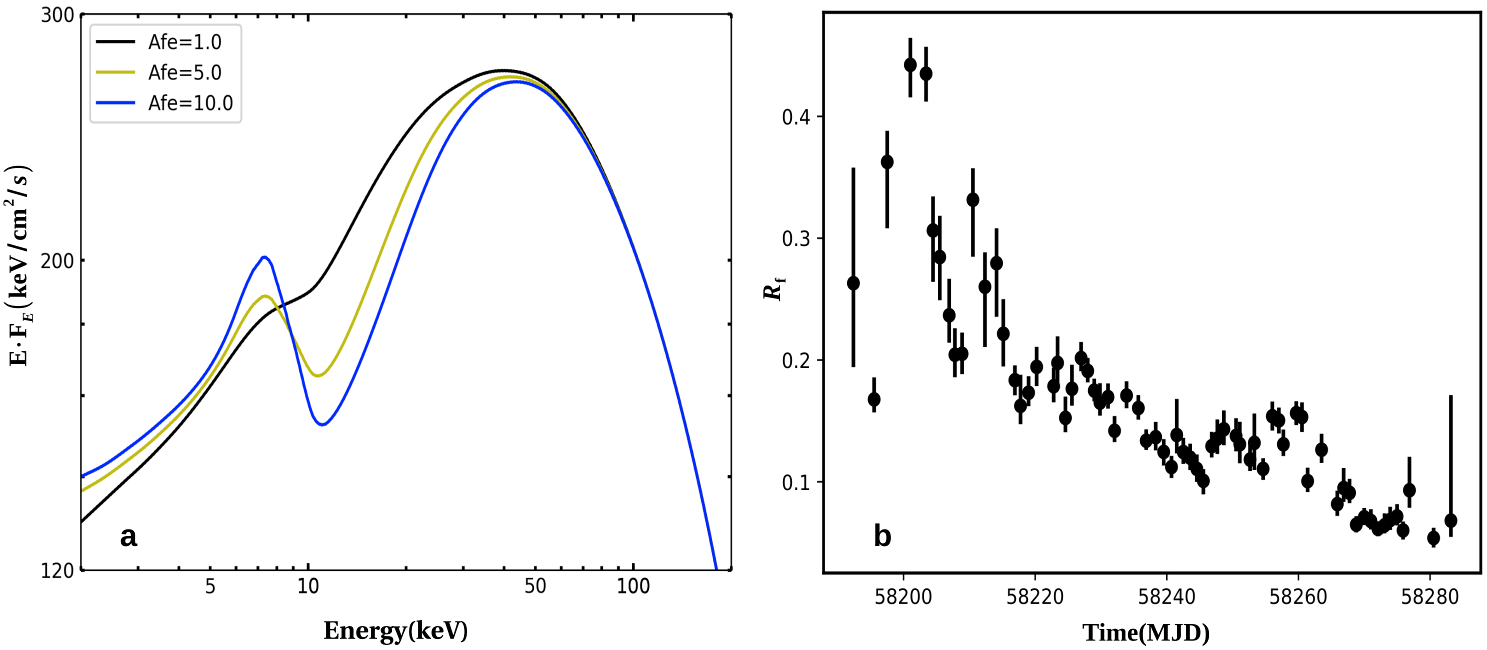

Supplementary Figure 4. (a): Relativistic reflection models in the HXMT bandpass using the relxillCp for the iron abundance $A_{\rm Fe}$ = 1.0, 5.0 and 10.0, in the case of the incident photon index $\Gamma = 1.7$ and the ionization parameter $\log\xi = 3.8$. **(b):** The best-fitting values of the reflection fraction $R_{\rm f}$, with the identical configuration of the fitting model in Fig. 3 of the main text, except fixing the iron abundance $A_{\rm Fe} = 5$. The black points correspond to the median of the values and the error bars correspond to 68% confidence interval, which is calculated using the corner[1] package to analyse the probability distributions derived from the MCMC chains. The uncertainties of the fitted parameters arise from both the statistical and systematic uncertainties.

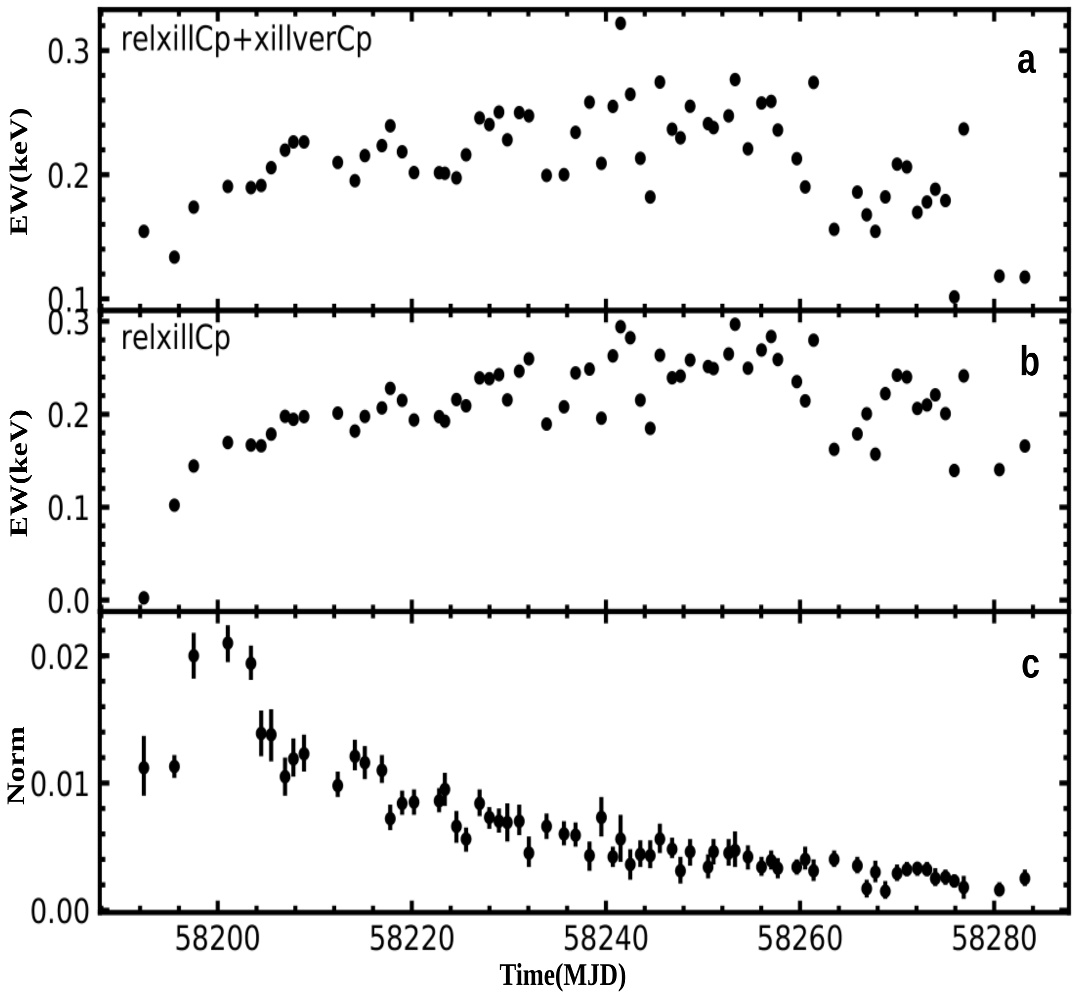

Supplementary Figure 5. **(a):** Time-evolutions of Equivalent width (in units of keV) of the broad Fe Kα line which is measured from the total model spectrum (relxillCp + xillverCp) in 4-10 keV; **(b):** Time-evolutions of Equivalent width of the broad Fe Kα line which is measured from the model spectrum of the relxillCp component; **(c):** Time-evolutions of the normalization of the xillverCp component. The black points correspond to the median of the values and the error bars correspond to 68% confidence interval, which is calculated using the corner[1] package to analyse the probability distributions derived from the MCMC chains. The uncertainties of the fitted parameters arise from both the statistical and systematic uncertainties.

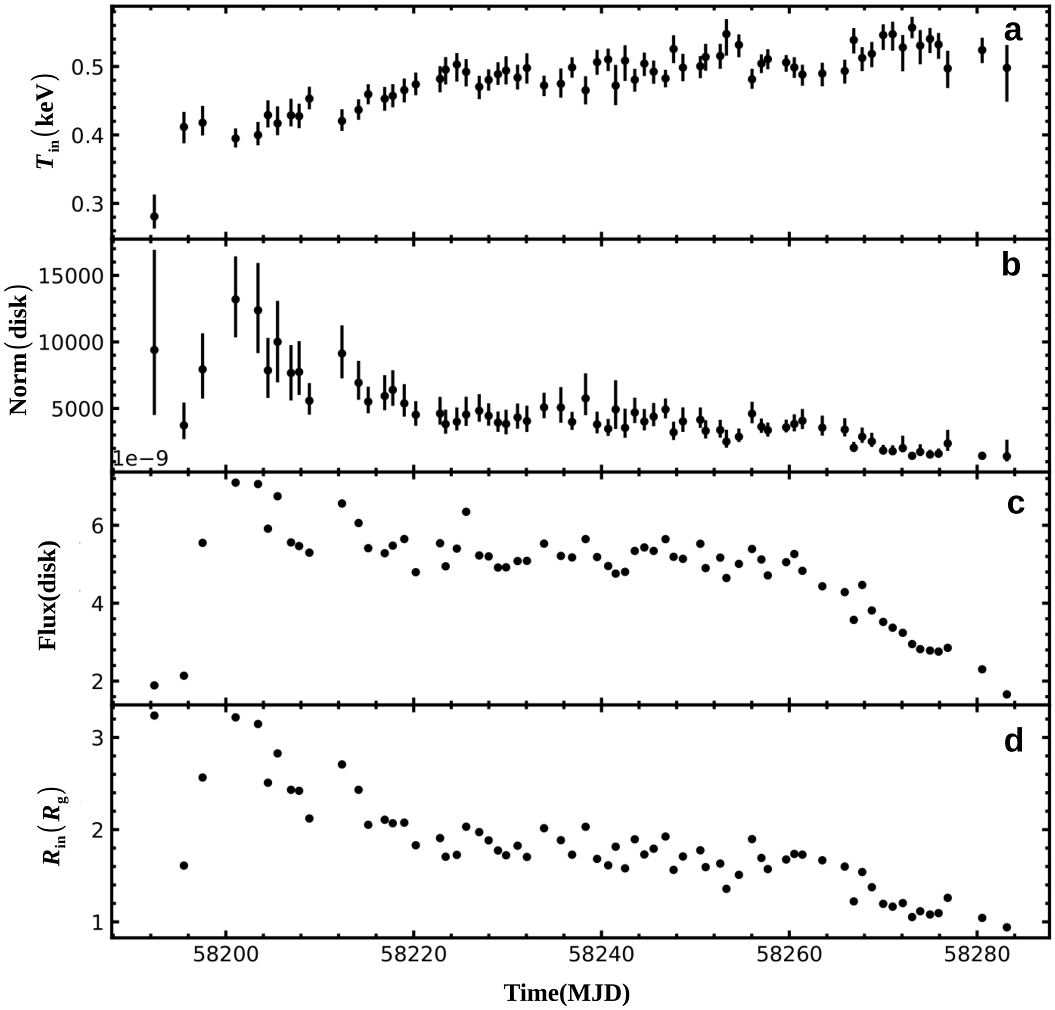

Supplementary Figure 6. Time-evolutions of the diskbb parameters, as plotted from top to bottom: the temperature at inner disk radius T_{in} in units of keV, the normalization, the diskbb flux in units of erg/cm^2/s, and the inner radius of the disk estimated from the diskbb photon flux. The black points in **(a)** and **(b)**, correspond to the median of the values and the error bars correspond to 68% confidence interval, which is calculated using the corner[1] package to analyse the probability distributions derived from the MCMC chains. The uncertainties of the fitted parameters arise from both the statistical and systematic uncertainties.

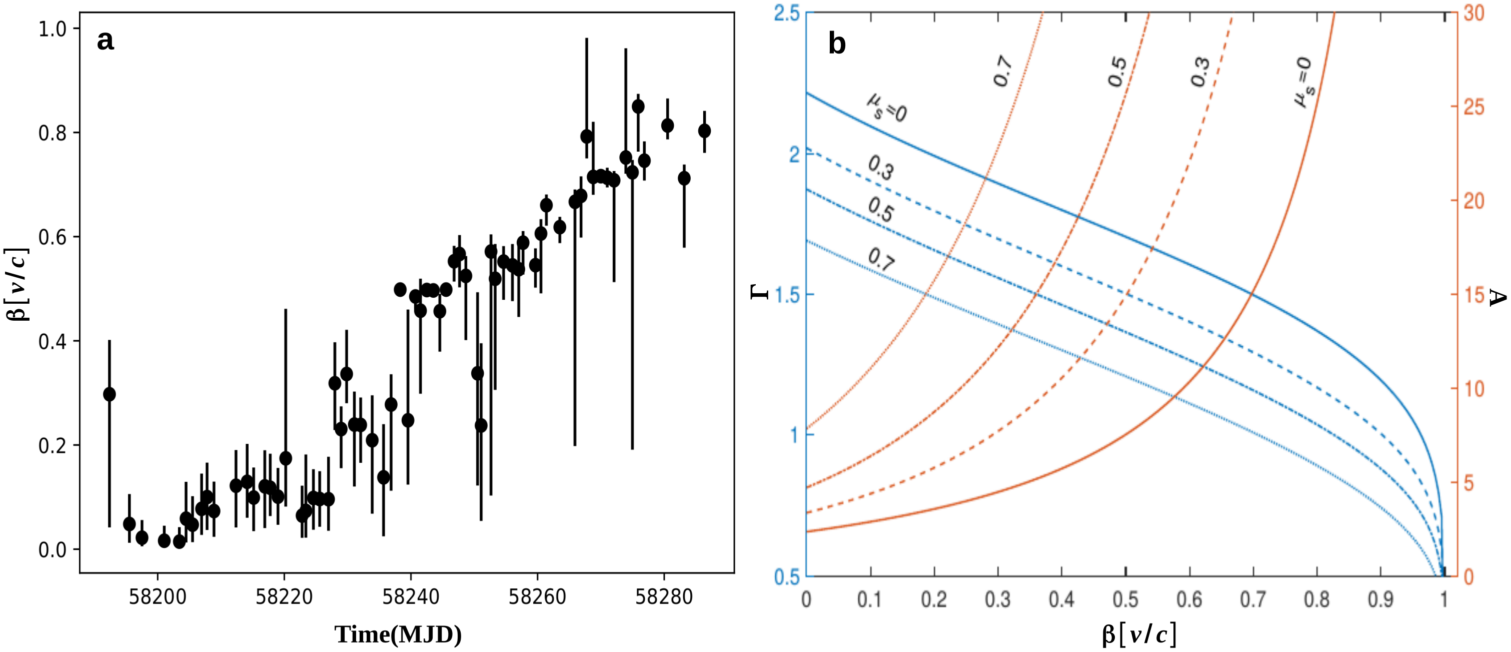

Supplementary Figure 7. (a): The outflowing velocities of the lamppost X-ray source in the best-fitting of TBabs∗(diskbb + relxilllpionCp + xillverCp)∗constant model. The new model relxilllpionCp (attributing to the reflection with the broad iron line) includes a velocity of the lamppost X-ray source which is set to be free here, and the height of the lamppost X-ray source is fixed at $H = 7R_g$. Assuming an inclination angle $\theta = 63^\circ$, the inner/outer radius of the reflection disk $R_{in} = R_{ISCO}$ (R_{ISCO} is innermost stable circular orbit) and $R_{out} = 1000R_g$, the black hole spin $a = 0.998$. The black points correspond to the median of the values and the error bars correspond to 68% confidence interval, which is calculated using the corner[1] package to analyse the probability distributions derived from the MCMC chains. The uncertainties of the fitted parameters arise from both the statistical and systematic uncertainties. **(b):** The geometrical factors μ_s-dependent Compton amplification factor A and the photon index Γ, as a function of outflowing velocity $\beta = v/c$, are plotted in red and blue line, respectively.

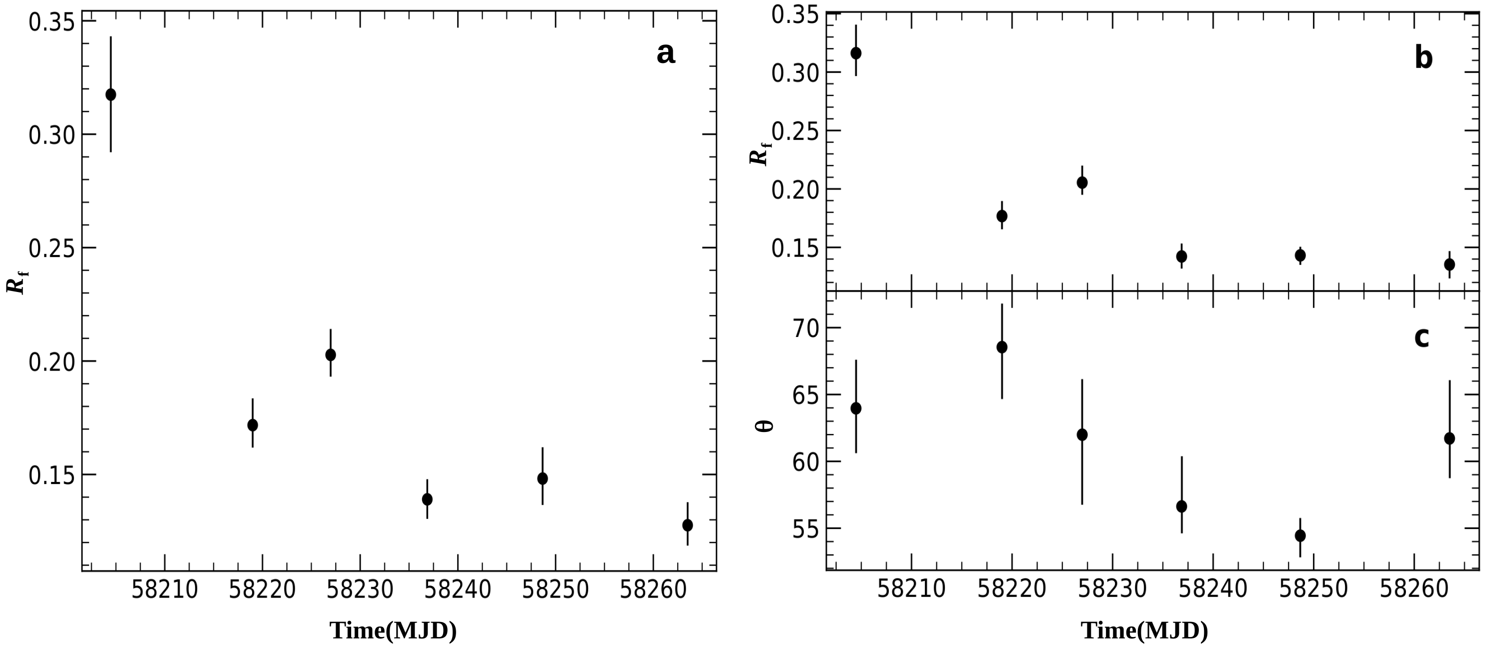

Supplementary Figure 8. (a): The reflection fraction for six epochs in the best-fitting of TBabs∗(diskbb + relxillCp + xillverCp)∗constant model, but the black hole spin $a = 0.5$. Assuming an inclination angle $\theta = 63°$. The reflection fraction **(b)** and the inclination angle **(c)** for six epochs in the best-fitting of TBabs∗(diskbb + relxillCp + xillverCp)∗constant model, where the inclination angle (degree) is a free parameter, but the spin is fixed at $a = 0.998$. Assuming the inner/outer radius of the reflection disk $R_{\rm in} = R_{\rm ISCO}$ ($R_{\rm ISCO}$ is innermost stable circular orbit) and $R_{\rm out} = 1000 R_{\rm g}$. The black points correspond to the median of the values and the error bars correspond to 68% confidence interval, which is calculated using the corner[1] package to analyse the probability distributions derived from the MCMC chains. The uncertainties of the fitted parameters arise from both the statistical and systematic uncertainties. The observation ID of six epochs are listed as follows: ObsID = P0114661006, P0114661017, P0114661024, P0114661032, P0114661043, P0114661055.

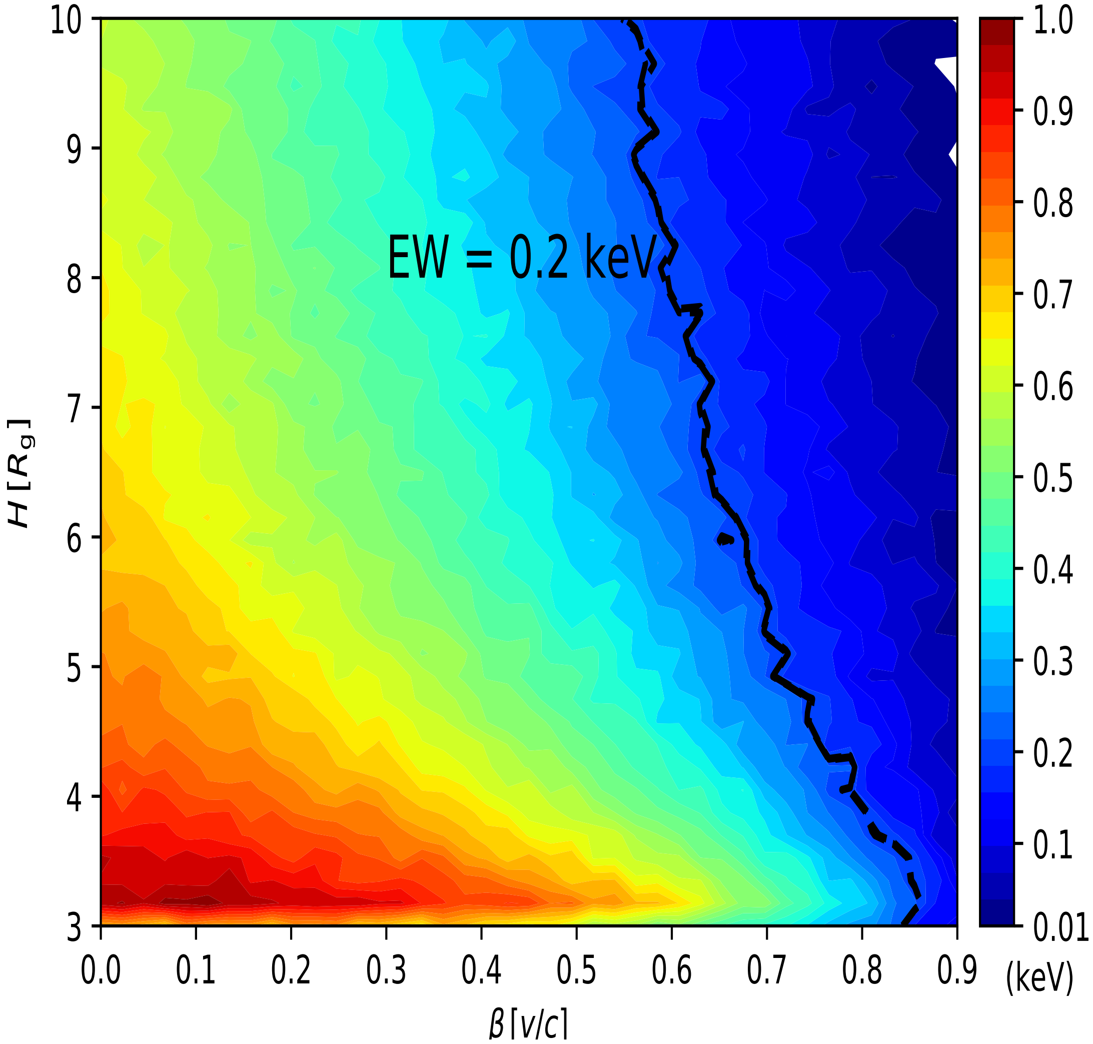

Supplementary Figure 9. Equivalent width (EW), in units of keV, as a function of the outflowing velocty ($\beta = v/c$) and the height, of the lower lamppost, while fixing the photon index $\Gamma = 1.55$. The EW is calculated with Equation (1), intergrating the reflection model spectrum by relxilllpionCp in 4-10 keV. The dashed line corresponds to the constant EW = 0.2 keV. The color bar represents the values of EW in units of keV, with the minimum and maximum being 0.01 and 1.0, respectively.

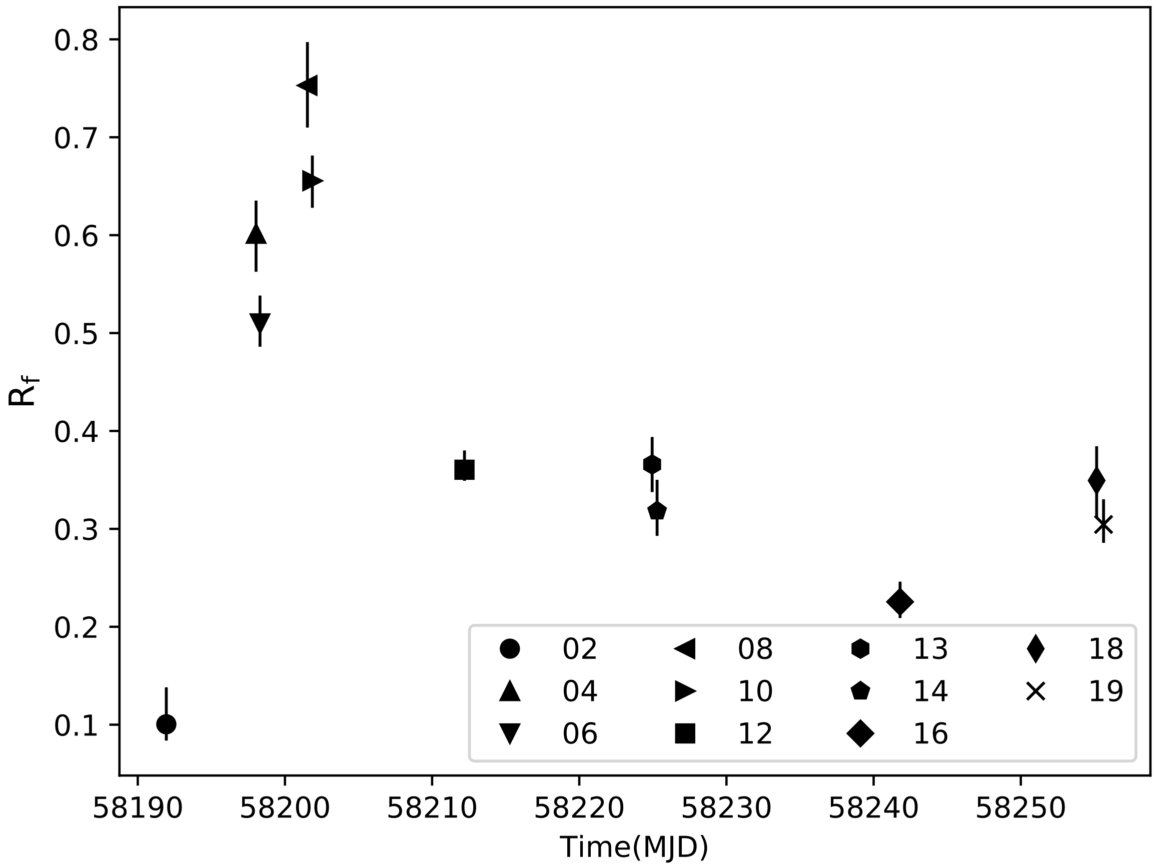

Supplementary Figure 10. The evolution of the reflection fractions of the relativistic reflection model relxillCp, which are estimated from the fits of NuSTAR spectrum. The numbers at lower right corner correspond to the obsID in Table 1 of ref.[2]. Note that, the first point (i.e., obsID = 02) is at the rise of MAXI 1820+070. For completeness, we still add it together with the rest points which roughly correspond to the decay of the outburst (see Fig. 1 of ref[2]). The data points correspond to the median of the values and the error bars correspond to 68% confidence interval, which is calculated using the corner[1] package to analyse the probability distributions derived from the MCMC chains. The uncertainties of the fitted parameters arise from both the statistical and systematic uncertainties.

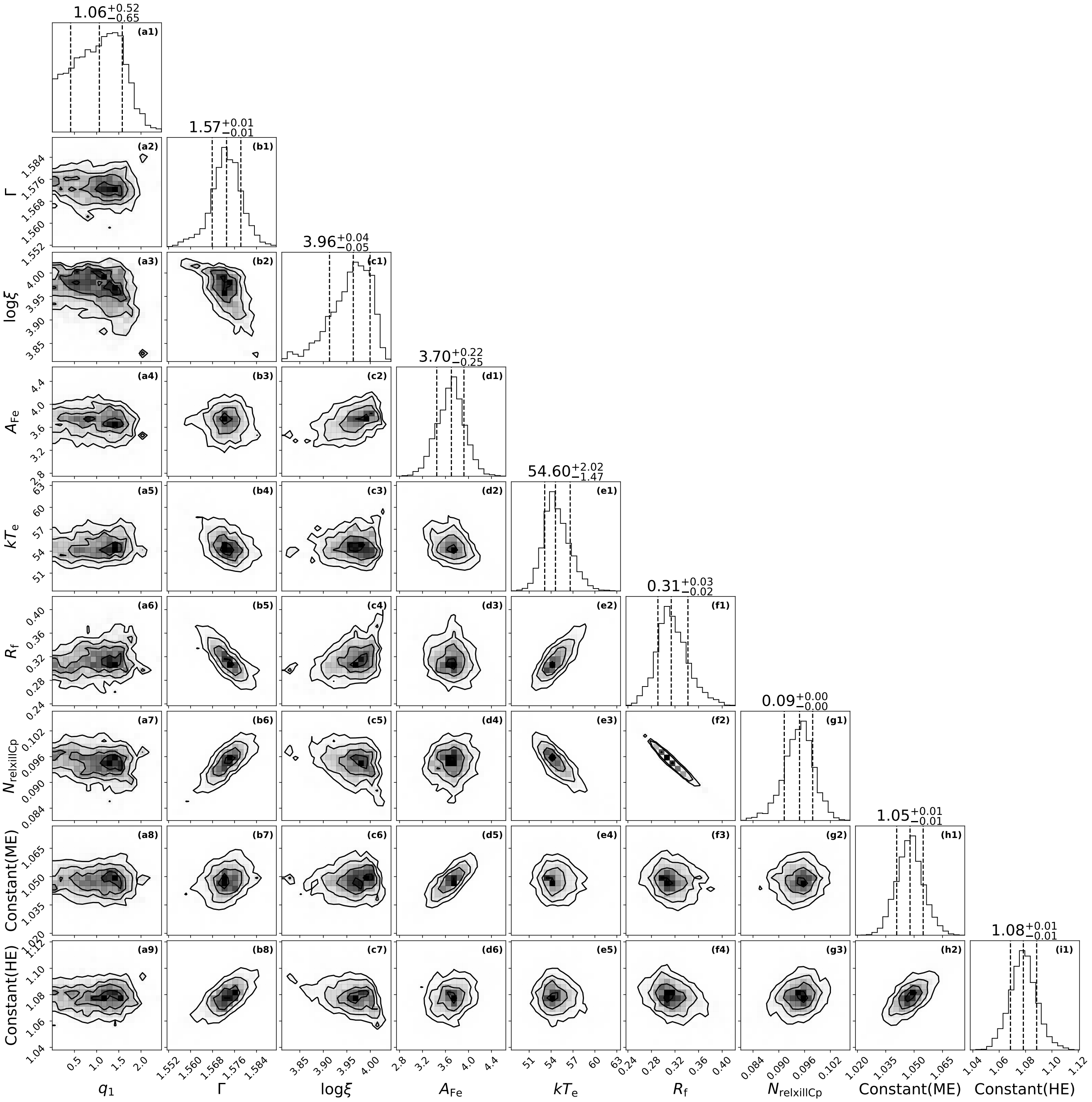

Supplementary Figure 11. An illustration of one and two dimensional projections of the posterior probability distributions derived from the MCMC analysis for the parameters in relxillCp, i.e., the emissivity profile q_1, the photon index Γ, the ionization parameter $\log\xi$ (in units of $\mathrm{erg\,cm\,s^{-1}}$), the abundance A_{Fe} with respect to the solar value, the electron temeperature kT_{e} (in units of keV), the reflection fraction R_{f}, the normalization, the constant for ME and the constant for HE. The contours in the two dimensional projections (the second to the bottom panel of each column) for each two parameters correspond to 1-, 2- and 3-σ confidence interval. The top nine panels (a1-i1) in each column are their one dimensional projections on the corresponding x axis. The values above each one dimensional projection indicate the median value of each parameter, as well as the upper and lower limits of the 68% confidence intervals. The vertical lines in the one dimensional projections correspond to the lower, median and upper value of each parameters. The MCMC analysis and the resultant figures above are produced using the corner package[1]. This illustration corresponds to the spectral fitting (see Fig. 1) of MJD 58204 (ObsID = P0114661006).

Component	Model	Parameter	OBSID			
			02	04	06	08
Soft flux	DISKBB	$\mathrm{Norm}_{\mathrm{FPMA}}$	$18.63^{+6.02}_{-3.21}$	$91.34^{+17.12}_{-17.64}$	$146.90^{+22.98}_{-18.35}$	$334.60^{+119.64}_{-57.02}$
		kT_{FPMA}/keV	$1.07^{+0.04}_{-0.06}$	$1.01^{+0.05}_{-0.04}$	$0.91^{+0.03}_{-0.03}$	$0.76^{+0.02}_{-0.03}$
	DISKBB	$\mathrm{Norm}_{\mathrm{FPMB}}$	$3.84^{+1.92}_{-0.70}$	$30.51^{+8.50}_{-6.09}$	$156.81^{+138.23}_{-42.08}$	$72053.81^{+20559.29}_{-22710.63}$
		kT_{FPMB}/keV	$1.34^{+0.06}_{-0.10}$	$1.17^{+0.07}_{-0.07}$	$0.81^{+0.05}_{-0.08}$	$0.35^{+0.01}_{-0.01}$
Compton continuum	NTHCOMP	$\Gamma_{\mathrm{FPMA,B}}$	$1.488^{+0.005}_{-0.015}$	$1.553^{+0.003}_{-0.003}$	$1.570^{+0.005}_{-0.004}$	$1.596^{+0.005}_{-0.004}$
		$kT_{\mathrm{FPMA,B}}$/keV	55^{+8}_{-5}	49^{+1}_{-1}	67^{+8}_{-6}	97^{+11}_{-12}
Disc	RELXILLCP	$R_{\mathrm{in}}/r_{\mathrm{ISCO}}$	1.0*	1.0*	1.0*	1.0*
		$\theta/^{\circ}$	63*	63*	63*	63*
		$A_{\mathrm{Fe}}/A_{\mathrm{Fe},\odot}$	$2.20^{+0.15}_{-0.14}$	$1.89^{+0.07}_{-0.07}$	$1.80^{+0.08}_{-0.12}$	$1.52^{+0.09}_{-0.08}$
Reflection	RELXILLCP	R_{f}	$0.10^{+0.04}_{-0.02}$	$0.60^{+0.03}_{-0.04}$	$0.51^{+0.03}_{-0.02}$	$0.75^{+0.04}_{-0.04}$
		$\log(\xi/\mathrm{erg\,cm\,s^{-1}})$	$4.05^{+0.12}_{-0.02}$	$3.87^{+0.02}_{-0.02}$	$3.82^{+0.01}_{-0.02}$	$3.80^{+0.02}_{-0.02}$
	XILLVERCP	R_{f}	−1*	−1*	−1*	−1*
		$\log(\xi/\mathrm{erg\,cm\,s^{-1}})$	1.0*	1.0*	1.0*	1.0*
	RELXILLCP	$\mathrm{Norm}_{\mathrm{FPMA,B}}$	$0.040^{+0.002}_{-0.004}$	$0.057^{+0.003}_{-0.002}$	$0.068^{+0.002}_{-0.002}$	$0.054^{+0.003}_{-0.002}$
	XILLVERCP	$\mathrm{Norm}_{\mathrm{FPMA,B}}$	$0.011^{+0.001}_{-0.001}$	$0.032^{+0.002}_{-0.002}$	$0.042^{+0.003}_{-0.003}$	$0.052^{+0.003}_{-0.003}$
	χ^2/d.o.f.		1.11	1.13	1.09	1.12

Supplementary Table 1. Best-fitting values to the NuSTAR spectra of MAXI J1820+070 in the hard state. The obsIDs: 904013090NN, correspond to the ones in Table. 1 of ref[2]. The model is TBabs∗(diskbb+relxillCp+xillverCp)∗constant. The asterisk indicates the parameters are fixed. The index of emissivity profile q_1 are pegged at zero, which are consistent with the spectral fits of HXMT. Errors represent 68% confidence intervals.

Component	Model	Parameter	OBSID			
			10	12	13	14
Soft flux	DISKBB	$\mathrm{Norm}_{\mathrm{FPMA}}$	$470.13^{+232.02}_{-89.41}$	$333.99^{+36.93}_{-29.94}$	$583.21^{+238.55}_{-106.09}$	$2275.17^{+883.40}_{-365.77}$
		kT_{FPMA}/keV	$0.75^{+0.03}_{-0.05}$	$0.80^{+0.01}_{-0.01}$	$0.74^{+0.03}_{-0.04}$	$0.58^{+0.01}_{-0.03}$
	DISKBB	$\mathrm{Norm}_{\mathrm{FPMB}}$	$711.13^{+449.45}_{-302.22}$	$1230.61^{+552.41}_{-209.17}$	$433.55^{+330.56}_{-133.15}$	$85285.85^{+10651.40}_{-13285.55}$
		kT_{FPMB}/keV	$0.62^{+0.05}_{-0.05}$	$0.59^{+0.01}_{-0.03}$	$0.72^{+0.05}_{-0.06}$	$0.35^{+0.01}_{-0.00}$
Compton continuum	NTHCOMP	$\Gamma_{\mathrm{FPMA,B}}$	$1.58^{+0.00}_{-0.00}$	$1.58^{+0.00}_{-0.00}$	$1.60^{+0.00}_{-0.01}$	$1.63^{+0.00}_{-0.00}$
		$kT_{\mathrm{FPMA,B}}$/keV	60^{+7}_{-6}	77^{+5}_{-4}	51^{+2}_{-2}	174^{+25}_{-24}
Disc	RELXILLCP	$R_{\mathrm{in}}/r_{\mathrm{ISCO}}$	1.0^{*}	1.0^{*}	1.0^{*}	1.0^{*}
		$\theta/^{\circ}$	63^{*}	63^{*}	63^{*}	63^{*}
		$A_{\mathrm{Fe}}/A_{\mathrm{Fe},\odot}$	$1.90^{+0.10}_{-0.09}$	$2.16^{+0.07}_{-0.06}$	$2.39^{+0.17}_{-0.13}$	$1.74^{+0.05}_{-0.06}$
Reflection	RELXILLCP	R_{f}	$0.66^{+0.03}_{-0.03}$	$0.36^{+0.02}_{-0.01}$	$0.37^{+0.03}_{-0.03}$	$0.32^{+0.03}_{-0.03}$
		$\log(\xi/\mathrm{erg\,cm\,s^{-1}})$	$3.83^{+0.01}_{-0.02}$	$3.81^{+0.01}_{-0.01}$	$3.87^{+0.03}_{-0.02}$	$3.71^{+0.00}_{-0.03}$
	XILLVERCP	R_{f}	-1^{*}	-1^{*}	-1^{*}	-1^{*}
		$\log(\xi/\mathrm{erg\,cm\,s^{-1}})$	1.0^{*}	1.0^{*}	1.0^{*}	1.0^{*}
	RELXILLCP	$\mathrm{Norm}_{\mathrm{FPMA,B}}$	$0.057^{+0.002}_{-0.001}$	$0.080^{+0.002}_{-0.002}$	$0.069^{+0.004}_{-0.003}$	$0.092^{+0.004}_{-0.005}$
	XILLVERCP	$\mathrm{Norm}_{\mathrm{FPMA,B}}$	$0.039^{+0.003}_{-0.003}$	$0.032^{+0.002}_{-0.001}$	$0.023^{+0.002}_{-0.002}$	$0.035^{+0.002}_{-0.001}$
		χ^2/d.o.f.	1.12	1.36	1.12	1.20

Supplementary Table 2. Best-fitting values to the NuSTAR spectra of MAXI J1820+070 in the hard state. The obsIDs: 904013090NN, correspond to the ones in Table. 1 of ref[2]. The model is TBabs∗(diskbb+relxillCp+xillverCp)∗constant. The asterisk indicates the parameters are fixed. The index of emissivity profile q_1 are pegged at zero, which are consistent with the spectral fits of HXMT. Errors represent 68% confidence intervals.

Component	Model	Parameter	OBSID		
			16	18	19
Soft flux	DISKBB	$\mathrm{Norm}_{\mathrm{FPMA}}$	$2080.77^{+260.61}_{-184.71}$	$1227.24^{+515.15}_{-183.10}$	$3867.70^{+1020.93}_{-558.61}$
		kT_{FPMA}/keV	$0.59^{+0.01}_{-0.01}$	$0.65^{+0.02}_{-0.03}$	$0.53^{+0.01}_{-0.02}$
	DISKBB	$\mathrm{Norm}_{\mathrm{FPMB}}$	$4360.92^{+1063.05}_{-1083.91}$	$1943.34^{+771.20}_{-594.78}$	$7828.79^{+2253.78}_{-1986.37}$
		kT_{FPMB}/keV	$0.49^{+0.02}_{-0.01}$	$0.58^{+0.03}_{-0.03}$	$0.45^{+0.01}_{-0.01}$
Compton continuum	NTHCOMP	$\Gamma_{\mathrm{FPMA,B}}$	$1.65^{+0.00}_{-0.00}$	$1.64^{+0.01}_{-0.01}$	$1.66^{+0.00}_{-0.00}$
		$kT_{\mathrm{FPMA,B}}$/keV	151^{+14}_{-15}	51^{+2}_{-2}	347^{+40}_{-61}
Disc	RELXILLCP	$R_{\mathrm{in}}/r_{\mathrm{ISCO}}$	1.0^{*}	1.0^{*}	1.0^{*}
		$\theta/^{\circ}$	63^{*}	63^{*}	63^{*}
Reflection	RELXILLCP	$A_{\mathrm{Fe}}/A_{\mathrm{Fe},\odot}$	$2.37^{+0.09}_{-0.08}$	$3.72^{+0.23}_{-0.37}$	$2.25^{+0.07}_{-0.05}$
		R_{f}	$0.23^{+0.02}_{-0.02}$	$0.35^{+0.04}_{-0.04}$	$0.30^{+0.03}_{-0.02}$
		$\log(\xi/\mathrm{erg\,cm\,s^{-1}})$	$3.72^{+0.01}_{-0.04}$	$4.03^{+0.02}_{-0.05}$	$3.73^{+0.00}_{-0.00}$
	XILLVERCP	R_{f}	-1^{*}	-1^{*}	-1^{*}
		$\log(\xi/\mathrm{erg\,cm\,s^{-1}})$	1.0^{*}	1.0^{*}	1.0^{*}
	RELXILLCP	$\mathrm{Norm}_{\mathrm{FPMA,B}}$	$0.090^{+0.003}_{-0.003}$	$0.052^{+0.003}_{-0.003}$	$0.070^{+0.002}_{-0.003}$
	XILLVERCP	$\mathrm{Norm}_{\mathrm{FPMA,B}}$	$0.018^{+0.001}_{-0.001}$	$0.010^{+0.001}_{-0.001}$	$0.015^{+0.001}_{-0.001}$
	χ^2/d.o.f.		1.17	1.17	1.14

Supplementary Table 3. Best-fitting values to the NuSTAR spectra of MAXI J1820+070 in the hard state. The obsIDs: 904013090NN, correspond to the ones in Table. 1 of ref[2]. The model is TBabs∗(diskbb+relxillCp+xillverCp)∗constant. The asterisk indicates the parameters are fixed. The index of emissivity profile q_1 are pegged at zero, which are consistent with the spectral fits of HXMT. Errors represent 68% confidence intervals.

References

1. Foreman-Mackey, D., Hogg, D. W., Lang, D., et al. 2013, PASP, 125, 306

2. Buisson, D. J. K., Fabian, A. C., Barret, D., et al. 2019, Mon. Not. R. Astron. Soc., 490, 1350
